# Social Capital as a Predictor of Quality of Life: The Czech Experience

**DOI:** 10.3390/ijerph19106185

**Published:** 2022-05-19

**Authors:** František Murgaš, František Petrovič, Anna Tirpáková

**Affiliations:** 1 Department of Geography, Technical University of Liberec, 461 17 Liberec, Czech Republic; fmtren@gmail.com; 2 Department of Ecology and Environmental Sciences, Constantine the Philosopher University in Nitra, 949 01 Nitra, Slovakia; 3 Department of Mathematics, Constantine the Philosopher University in Nitra, 949 01 Nitra, Slovakia; atirpakova@ukf.sk

**Keywords:** quality of life, social capital, predictor, trust, Czechia

## Abstract

In the paper, we understand social capital as a variable that affects the quality of life. A variable whose change affects another variable is called a predictor. The paper is based on Putnam’s understanding of social capital with the dimensions of trust, norms and networks. Trust is considered the most important dimension, and for the purposes of the paper social capital is identified with trust. Quality of life is a holistic concept with two dimensions expressing an assessment of satisfaction with life. After society became richer—in the 1960’s in the West and, after the collapse of the bipolar world, also in Central and Eastern Europe—the need for quantity was replaced by the need for quality. The paper is focused on Czechia, with social capital as a predictor of quality of life being investigated geographically at the level of districts. According to the research hypothesis, social capital will have a strong influence on the quality of life of residents in Czechia, i.e., it will be its predictor. To test the validity of the research hypothesis, research was conducted. The aim of the paper is to outline the epistemology of social capital from the aspect of quality of life, description of quality of life and then to test the validity of the research hypothesis by measurements. The result of the quantification of social capital and quality of life at the level of districts and their correlation is important from an epistemological point of view for two reasons. The first is to question the generally accepted premise of the position of social capital as a strong predictor of quality of life. The second is the recognition that the premise of the position of social capital as a strong predictor of quality of life applies in the districts with the highest quality of life.

## 1. Introduction

Throughout history, people have been asking themselves how to live, what kind of life will bring them contentment. For centuries, religion has provided the answers to the question of the meaning of human existence and the kind of life people will be satisfied with. The Enlightenment brought secularization to the people of the West, which greatly diminished the influence of religion on society, but interest in the good life did not diminish. The unprecedented growth of prosperity after the Second World War in the USA and the countries of Western Europe and, after the collapse of the bipolar world, in the countries of Central and Eastern Europe as well, greatly raised the standard of living of the inhabitants. The fulfilment of the need for “quantity” of life in the sense of satiation of material needs has led to increasing interest in “quality” of life. Scholars have formulated the concept of quality of life, which has become a common part of public policy and public debates. According to Mularska-Kucharek [1], quality of life has become “a sign of the spirit of the times”.

Quality of life, sometimes identified with life satisfaction, well-being or happiness, is influenced by many variables, one of which is social capital. This statement is generally accepted not only by scholars focused on quality of life but also by international institutions such as the OECD or the World Bank. A variable the change of which affects another variable is understood as a “predictor”. Cambridge Dictionary [2] defines a predictor as “something such as an event or fact that enables you to say what will happen in the future”. Predictors of quality of life have been addressed by several authors [3,4,5]. Variables affecting quality of life are also commonly referred to as “drivers of quality of life” [6,7]. In the paper, the variable affecting the quality of life is called a *predictor*.

In the paper, we focus on social capital as a predictor of quality of life. We understand the term predictor as an influencing variable which is in a relationship with another, influenced variable. A change in the value of the influencing variable will be reflected by a change in the influenced variable. The relationship between the terms “predictor” and “quality of life” has two different levels. In our case, the first one expresses the influence of social capital as a predictor, i.e., the influencing variable, on the influenced variable, which is the quality of life. Such a variable can include age, education [8] or social support [9]. Mrva [10] considers personal trust as a predictor of quality of life in Slovakia. We note that Slovakia was part of Czechoslovakia together with Czechia until the end of 1992.

The second level is formed by quality of life, which is an influencing variable and as such a predictor of some influenced variables. Quality of life is a predictor of health [11,12,13], depression [14] and the outcome of the Czech presidential election [15]. Predictors of quality of life need to be distinguished from factors supporting its improvement [16].

Social capital is considered a strong predictor of quality of life in the sense that the higher the values of social capital, the higher the values of quality of life. According to Rezaei Niaraki [17], it is “one of the key determinants”. This statement is expressed by authors from countries considered individualistic [1,18,19,20] and collectivistic [21,22]. According to Bartolini et al. [23], the decline of social capital in American society has caused a process of stagnation in the quality of life despite the growth of prosperity, which has become known as the Easterlin paradox. In the U.S., there has been a decline in the level of happiness over the past three decades, accompanied by a decline in the level of both social ties and institutional trust. Bartolini et al. [24] justify this by arguing that both social ties and institutional trust are predictors of happiness, and therefore, a decline in one variable (social ties and trust) is accompanied by a decline in the other variable (happiness). Sirgy [25] explains such changes by the theory of “horizontal spillovers”. We consider the claims of Bartolini et al. [23,24] as serious, and therefore, we focus on the relationship between social capital and quality of life in Czechia. In quality research, this country shows results as different from the mainstream [15,26], which has increased interest in exploring the relationship between social capital and quality of life, formulated in the hypothesis. The result will provide valid information for public policy in Czechia.

In the paper, we deal with social capital as one of the intangible and non-financial capitals of a person and its relation to the quality of life, expressing the degree of satisfaction with life. We look at social capital, trust and quality of life from a geographical perspective, and we are interested in their spatial differentiation. We understand quality of life holistically in terms of its two dimensions. As such, the following is our research Hypothesis 1 (H1). To test the validity of the research hypothesis, research was conducted. The aim of the research was to outline the epistemology of social capital in terms of quality of life and the description of quality of life, and then to test the validity of the research hypothesis.

**Hypothesis 1** **(H1).** *Social capital will have a strong influence on the quality of life of residents in Czechia, i.e., it will be a predictor of the quality of life.*

## 2. Theoretical Background

Social capital is one of the capitals of a person; it differs from other capitals in that it is relational. The fact that it is based on relationships between people implies that it is not the property of the individual but represents a public good. The relationships between people which generate social capital are strongly influenced by their cultural and axiological anchoring and accepted norms [27]. Kučerová [28] highlighted that the size or quantity of social capital is determined by two factors—the characteristics of social networks (number of members, level of trust) and the quality of the place in which social interactions take place. Quality of life is a concept by which people express their level of satisfaction or dissatisfaction with their lives. It has two dimensions, a subjective one, referred to by the term well-being, and an objective one, conceptualized as quality of place [29]. It follows that social capital and quality of life are linked by a common element, which is the place. For scholars focused on quality of life, one of its aspects is considered crucial, and this is the place-formed quality of conditions for a good life.

### 2.1. Social Capital

Social capital is an interdisciplinary concept that has received a great deal of attention, particularly in the social sciences. Social capital has positive effects on physical and mental health, happiness, education, crime, welfare, community relations, life expectancy and public policy. Relationships are the basis of social capital; relationships between people are strongly influenced by their cultural and axiological anchoring and accepted norms. The dimensions of social capital include trust, norms and networks [30].

**Figure 1:**
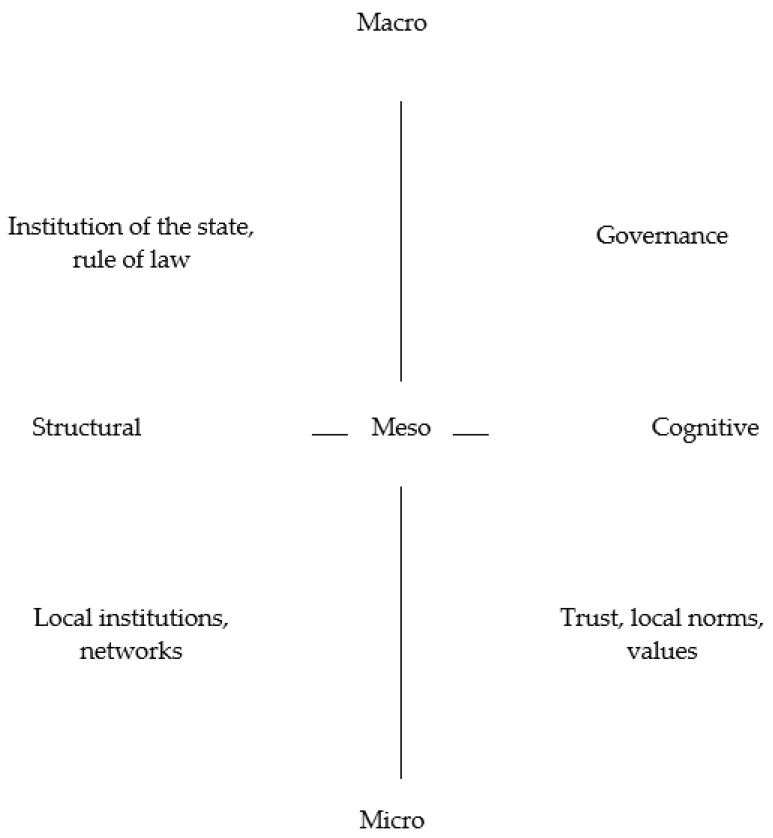
The Forms and Scope of Social Capital [35].

We consider trust to be the most important component of social capital; in the paper, we identify social capital with trust. According to Putnam [30], “social capital and trust belong together and are inseparable”. Measuring personal trust has “fundamental importance to assessing the well-being of societies, to measuring social capital, and to understanding the drivers of other social and economic outcomes”, as highlighted by the OECD [31]. “The academic research on trust has highlighted a number of relations between trust and a range of outcomes that matter for the well-being of people and of the country in which they live”, as highlighted by Algan [32]. The model of social capital research as a predictor of quality of life is shown in Figure 1.

As is the case with other contemporary concepts, social capital does not have a formalized epistemology, and so there is no general consensus on whether trust is an output of social capital (defined as networks, shared values and norms) or, conversely, social capital is the joint output of trust, shared values and norms that constitute social capital [27].

The “founding fathers” of the concept of social capital are Pierre Bourdieu, Robert Putnam and James Coleman. The scholar who has contributed most to the popularization of the concept of social capital is Putnam [30]. Although social capital was mentioned as early as the 1960s by Jane Jacobs in her well-known work *The Death and Life of Great American Cities* [33], social capital was first conceptualized by Pierre Bourdieu, who considered it as one of three capitals; in addition to social capital, these include economic capital and cultural capital. It is also generally known that social capital is divided into structural and cognitive capital, represented by trust [34]. Grootaert, van Bastelaer [35] in their structure of social capital (Figure 1), supplement its division into structural and cognitive with three spatial levels.

There are many definitions of social capital, many of which share common elements of social networks, norms, values and trust. One of the most well-known is the following definition that social capital is formed by “features of social organization such as networks, norms, and social trust that facilitate coordination and cooperation for mutual benefit” [36]. The OECD [27] highlighted that social capital is formed by “networks together with shared norms, values and understandings that facilitate co-operation within or among groups”. In social capital, according to Mularska-Kucharek [1], “communication and participation networks, trust and values” are essential. Such a conceptualization of social capital is close to Putnam’s approach, and it is accepted especially by authors focused on empirical research of social capital [37]. A review of definitions of social capital is presented by Mularska-Kucharek [1].

There is no consensus among social scientists as to whether social capital operates at the individual level, the collective level, or both [38]. According to Mularska-Kucharek [1], “both individual and collective social capital is necessary for high quality of life”. Bourdieu understood social capital at the individual level, Putnam at the collective level. Related to this is the lack of consensus on whether social capital is a private good, private-public good or public good. The concept of social capital has its critics, with some stating that it is too disparate in definition, application and problematic in the sources used for measurement [39].

One of the key components of social capital—an influencing variable of quality of life—is trust. According to Theurer, Wister [40] its impact on quality of life is robust. In the paper, we understand it as an emotion manifested in interpersonal relationships. Cambridge Dictionary [41] defines trust as follows: “to believe that someone is good and honest and will not harm you, or that something is safe and reliable”. Trust comes in two forms—on the one hand, personal trust in other people, and on the other hand, trust in institutions such as local government, the police, churches or elected bodies from local government to the European Parliament. The second type of trust is called institutional or generalized trust [18]. Parks et al. [42] state that this trust is a “public good”. Trust, both personal and generalized, is one of the three dimensions of social capital. Other dimensions include social networks and social norms [43]. Eriksson [44] considers social capital as a crucial part of social sustainability.

GIS tools are suitable for the spatial representation of regional disparities, as confirmed by the work of several authors [45,46,47]. In the paper, we focus on the impact of social capital on quality of life. Another topic is the study of the impact of quality of life on social capital. Onyx, Bullen [48] consider it for one of the eight indicators of social capital.

#### 2.1.1. Linking Social Capital and Quality of Life

The links between social capital and quality of life have been addressed by several authors [49,50]. Algan [31] reports a very high correlation of 0.77 between social capital and life satisfaction. Social capital in conjunction with quality of life is investigated in a wide range of focuses, ranging from pregnant women [17] to migrants [51]. Social capital in relation to health-related quality of life is frequently investigated [52]. The OECD [26] explores the relationship between social capital and well-being. Social capital in the long run is a major predictor of quality of life [53].

Social capital also relates to the dilemma of the validity or invalidity of Easterlin’s paradox. Some authors support it and quantify their claims, while others reject it and quantify their claims as well. The dilemma could be resolved by recognizing that welfare growth improves the quality of life if income inequality falls and personal trust rises at the same time [51,52,53,54,55,56].

#### 2.1.2. Development of Social Capital in Czechia

**Figure 2:**
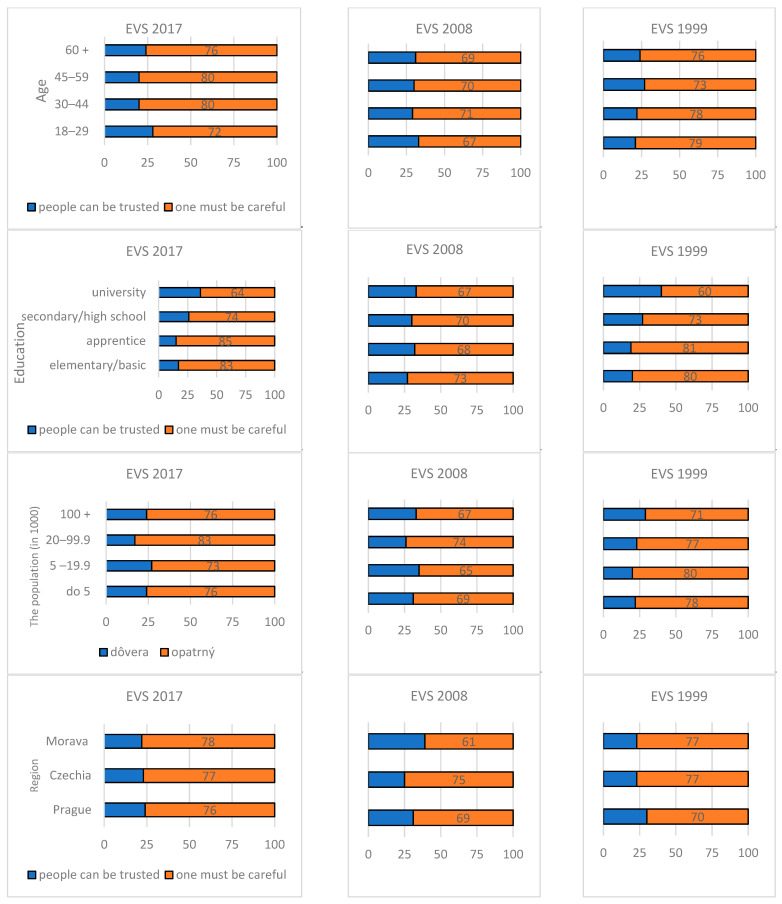
The development of social capital.

For the examination of the development of social capital in Czechia, one can use the data collected for the European Values Study (hereinafter referred to as EVS), specifically the data on personal trust (Table 1, Figure 2). The EVS survey was conducted in 1999, 2008 and 2017; the number of respondents over 18 years of age ranged from 1812 in 2017 to 2109 in 1991 [57].

A comparison of trust in 2017 and 1999 shows that, of the 18 categories, trust in 2017 was lower in 13 categories, including the “total” category, higher in 4 categories and the same in 1 category. There was a decline in trust for university-educated people (down 7%), for people living in large cities (by 5%) and in Prague (by 8%).

### 2.2. Quality of Life

Quality of life is one of the concepts by which late modern society tries to understand the complexity of the contemporary world. The term “quality of life” expresses the emotional and cognitive evaluation of one’s own life. When we evaluate our life, we evaluate our satisfaction with it. Quality of life is most often measured on a Cantril scale of 0–10. The expression of satisfaction is well-being, the expression of dissatisfaction is ill-being. In developed countries, satisfaction dominates in the evaluation (on the Cantril scale values 6–10), which is why some scholars identify quality of life with well-being. The structure of quality of life, its dimensions, domains and indicators were highlighted by Petrovič, Murgaš [58]. One of the many explanations for the growing interest in quality of life is based on the assumption that quality of life is the result of the search for meaning and form of life in the contemporary world. “One would risk the affirmation that category of quality of life is a sign of the spirit of the times or an idea for which the time is now” [1]. The need to develop an epistemology of quality of life is understood by Macků et al. [59] as a necessary condition for the ability to produce valid evidence for public policy. Quality of life in the Czechia has been addressed by several authors [15,26,29,59,60,61,62,63].

### 2.3. The Geographical Dimension of the Relationship between Social Capital and Quality of Life

Both social capital and quality of life receive attention in the field of geography [64,65,66,67,68,69,70]. In countries and regions, social capital and quality of life do not look homogeneous because they have a strong geographical dimension. Following Putnam’s pioneering study looking at the evolution of social capital in the US [29], other authors [19,37,71,72] have begun to study social capital at the country level. Sarracino [19] examined social capital and its impact on quality of life in eleven Western European countries between 1980 and 2000 and found that social capital is growing in these countries, in contrast to the US where it is eroding. In Europe, however, one country differs from the others—Great Britain, where social capital is declining. Sarracino, Mikucka [73] looked at the evolution of the components of social capital in European macro-regions. They investigated the highest levels in Scandinavia and the Netherlands and in Southern Europe. In Central and Eastern Europe, the level is the lowest, but the region is very heterogeneous. Slovenia, Czechia and Slovakia have values close to the European average, while Poland and Hungary have lower values. Social capital is lowest in the countries of the former Soviet Union, especially Russia, Ukraine and Belarus. Across Europe, trust has been rising; of the other components of social capital, some have been rising, while others have been eroding.

Based on an analysis of trust in 46 countries, Mikucka et al. [55] found that growth in personal trust correlates more strongly with quality of life in developed countries compared to emerging countries. In rich developed countries, income inequality generally declines, which in turn boosts trust growth.

Another finding is that economic growth does not correlate with quality of life in countries without transformation. This confirms Easterlin’s observation that although people in richer countries are happier on average, they are not more satisfied. However, the observation of growing GDP without increase in quality of life does not hold for transition countries where quality of life is positively and significantly correlated with economic growth. Hence, transition countries are an exception to Easterlin’s paradox [74]. Scholars from the West consider all former socialist countries as one group, which they call Eastern Europe, but this does not correspond to reality. Scholars from Czechia, Slovakia, Poland and Hungary refer to their countries as Central Europe and the former republics of the Soviet Union as Eastern Europe. These four countries are post-transition countries, and Czechia, as the most economically successful of them, has surpassed Greece or Portugal in some indicators. Specifics of the quality of life in Czechia have been explored by Murgaš [15].

Japan has a special position in the study of quality of life as a country with the highest life expectancy in the world. At the same time, Japan has low levels of subjective well-being, happiness and personal trust compared to other countries [75].

## 3. Methodology

Our study of social capital as a predictor of quality of life is divided into three chapters. In the first chapter, we focused on the theoretical background of the study of social capital as a predictor of quality of life. In the second chapter, we focused on the description of data acquisition and the measurement of social capital and quality of life. In the third chapter, we discuss the knowledge gained.

Methodologically, we proceed in several steps. The first step is justification of research, aim formulation and hypotheses. The second step is reviewing the literature. The third is the analysis of the development of social capital in the Czech Republic and the analysis of the development of quality of life. The fourth step is measuring the social capital using the indicator ‘personal confidence’ and the quality-of-life indicator ‘overall quality of life’. The measured values are illustrated with images in the form of maps of Czech districts, created using the ArcGIS Pro 2.9.0 software (Esri, 380 New York Street, Redlands, CA, USA). The fifth step is discussion, stating the validity/invalidity of the hypothesis and formulation of the Conclusion.

The evolution of the quality of life in the regions of Czechia in the period 2003–2015 has been discussed by Murgaš [15]. In his assessment of that period, he finds stagnation in the development of quality of life. The Czech Statistical Office (CSO) surveyed the quality of life in 2013 [76] and 2018 [77]. In 2013, respondents older than 16 years rated their satisfaction on a scale of 0–10. The results showed a life satisfaction to be 6.9 for males and 7.0 for females. Nearly 25% of respondents expressed their satisfaction with a value of 8. The fact that only 10% of the respondents reported a satisfaction value lower than 5 is indicative of the level of satisfaction.

The life satisfaction survey was repeated by the CSO in 2019 under the same conditions, i.e., on a 0–10 scale for residents aged 16 years and older. Compared to 2013, satisfaction with life increased to 7.4, for both men and women. The number of respondents who reported a value of their satisfaction of less than 5 has decreased from 10% in 2013 to 5% in 2018. For the unemployed who reported the lowest satisfaction level, their average satisfaction value rose from 5.4 in 2013 to 6.3 in 2018, and for pensioners with the second lowest satisfaction level, the value rose from 6.5 in 2013 to 6.9 in 2018. At the other end of the economic activity spectrum—students reporting the highest level of life satisfaction—the value rose from 8.0 in 2013 to 8.2 in 2018. Two important findings emerge from comparing the 2013 and 2018 CSO life satisfaction survey across regions: (i) satisfaction is rising across all categories, and (ii) spatial patterns in 2018 are significantly different from spatial patterns in 2013.

The growth of satisfaction with life in the Czechia is also confirmed by a measurement carried out by the Public Opinion Research Centre at the Institute of Sociology of the Academy of Sciences (hereinafter referred to as CVVM). The survey has been conducted since 1996, and respondents answer the question “How satisfied are you with your life overall?”. Respondents have six options: (i) very satisfied, (ii) somewhat satisfied, (iii) neither satisfied nor dissatisfied, (iv) somewhat dissatisfied, (v) very dissatisfied, and (vi) do not know [78].

The analysis of the development of quality of life in the Czechia between the years 1996 and 2020 shows long-term trends:

- At the beginning of the measurement in December 1995, the variable satisfaction with life had a value of 55.7%, neither satisfaction nor dissatisfaction had a value of 32.3%, the variable dissatisfaction had a value of 11.7%, and the variable do not know had a value of 0.3%.
- Between 1996 and 2002, the values for satisfaction, neither satisfaction nor dissatisfaction and dissatisfaction were stagnant.
- In the period 2002–2003, the satisfaction value increased (February 2003—satisfaction 59.8%), the values of neither satisfaction nor dissatisfaction decreased (February 2003—25.8%) and oscillated around 25% until 2020, and the dissatisfaction value increased slightly (February 2003—14.2%) and oscillated around 15% until 2012.
- In 2012, the satisfaction value started to rise, from a value of 51.2% (October 2012) to a value of 69.3% in February 2020. The dissatisfaction value rose to 21.6% in 2012–2013 (October 2013), but since then it has been decreasing.
- In 2020, the satisfaction variable had a value of 69.1%, neither satisfaction nor dissatisfaction a value of 22.4%, the dissatisfaction variable a value of 8.3% and the do not know variable a value of 0.2%.

The vagueness of the term social capital implies a number of indicators used in its measurement. When examining social capital as a predictor of quality of life, the indicators used included the following: trust, social relations, commitment, communication, influence [18], social participation, social network, trust and reciprocity [51].

We tested the validity of the research hypothesis by conducting research at the spatial level of Czech districts. Respondents over 18 years of age (N = 1356) participated in the research, using the face-to-face method and social networks. Respondents expressed the values of quality of life and trust on scale 0–10. Data were collected from all 77 districts of Czechia so as to meet the district quota selection criterion (in the statistical designation LAU 1). Adequate representation of all districts was the primary goal. The secondary goal was to strive for a balanced number of men and women and to represent all age groups. Respondents answered the following question: “Indicate on a scale of 0–10 how much you trust people. 0 means you do not trust anyone and 10 means you trust everyone”. For quality of life, the question was: “What is your overall quality of life? Please indicate on a scale of 0–10, where 0 is the worst possible and 10 is the best possible”.

Based on the data obtained, we divided the districts of Czechia according to the calculated average values of the quality-of-life indices into the following three groups: group 1—up to 6.99; group 2—from 7.00 to 7.99; and group 3—districts above 8.00. The second observed feature, according to which we also divided the districts of Czechia into three groups, was the calculated mean values of the indices characterizing personal trust. These included the following groups of districts of Czechia: group 1 consisted of districts where the average value of the personal trust index was calculated up to 5.49. The second group included districts where the average personal trust index values were between 5.5 and 6.49, and the third group included districts with personal trust index values above 6.5.

An appropriate statistical method is usually used to verify the validity of the research hypothesis. In our case, we used selected statistical methods to verify the dependence of two qualitative features *A*, *B*, specifically by the chi-square test of independence for the type of contingency table [79].

It is assumed that character *A* acquires *k* levels $A_{1},A_{2},\ldots ,A_{k}$ and character *B* acquires *m* levels $B_{1},B_{2},\ldots ,B_{m}$, whereby k>2 or m>2. Character *A* represents groups of districts formed on the basis of calculated average values of quality-of-life indices, and character *B* represents groups of districts formed on the basis of calculated average values of indices characterizing personal trust. We tested the null hypothesis $H_{0}$ that characters *A*, *B* are independent against the alternative hypothesis $H_{1}$ that characters *A*, *B* are dependent. As a test criterion, we used the chi-square statistic which is given by the relationship

$$\chi^{2}=\sum_{i=1}^{k}\sum_{j=1}^{m}\frac{{\left(f_{ij}-o_{ij}\right)}^{2}}{o_{ij}}$$

where $f_{ij}$ are empirical abundances and $o_{ij}$ are expected abundances. The chi-square test statistic holds for the validity of the hypothesis being tested $H_{0}$. $\chi^{2}$-distribution with number of degrees of freedom *r* =(k−1)(m−1). We reject the tested hypothesis $H_{0}$ at the significance level α, if the value of the test criterion $\chi^{2}$ exceeds the critical value $\chi_{\alpha }^{2}(r)$. The critical value $\chi_{\alpha }^{2}(r)$ can be found in the chi-square distribution critical value table.

Thus, the chi-square test of independence for the contingency table k×m was used to test whether the value of the personal trust index was related to the value of the quality-of-life index. We conducted the test using the STATISTICA program. After entering the input data in the computer output report, we obtained the contingency table (Table 2, Figure 2), the value of the chi-square test criterion and the probability *p*-value.

We can also evaluate the test using the *p*-value, which is the probability of error we make when we reject the hypothesis being tested. If the *p*-value is sufficiently low (*p* < 0.05 or *p* < 0.01), we reject the tested hypothesis $H_{0}$ of the independence of the observed characters A, B (at the significance level of 0.05 or 0.01). Otherwise, we cannot reject the hypothesis $H_{0}$. Since in our case the calculated probability *p*-value is greater than 0.05 (*p* = 0.100), we cannot reject the hypothesis $H_{0}$ of independence of the observed characters at the significance level α=0.05, i.e., the validity of the research hypothesis *H*_1_ is not confirmed.

**Figure 3:**
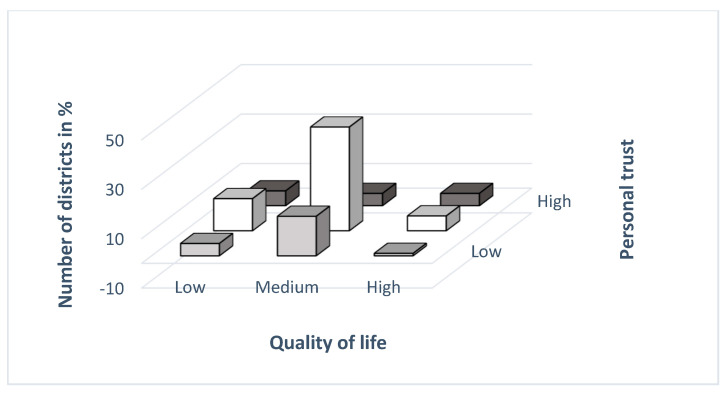
Distribution of number of districts according to average values of quality of life and personal trust (in %).

This means that social capital at the district level does not have a strong impact on, or is not a predictor of, the quality of life of residents of Czechia. In other words, the personal trust values of respondents in the districts of Czechia do not affect the quality-of-life evaluation. We illustrate the above statement in Figure 3, where we see that the number of districts (expressed in %) in the groups according to the values of quality of life and in the groups pointed to the values of personal trust are different. The distribution of the number of observed characters in % is specified in Table 3.

## 4. Results and Discussion

Table 3 shows that the largest number of districts—up to 42%—belong to the second (medium) group both according to the values of personal trust indices and according to the quality-of-life values.

At the end of the statistical analysis of the research results, we calculated the degree of dependence between the two quantitative characters: between personal trust and quality of life using the contingency coefficient [79]. The contingency coefficient is defined by the following formula.

$$C=\sqrt{\frac{\chi^{2}}{n+\chi^{2}}},\mathrm{where}\chi^{2}=\sum_{i=1}^{k}\sum_{j=1}^{m}\frac{{\left(f_{ij}-\frac{f_{i}^{A}f_{j}^{B}}{n}\right)}^{2}}{\frac{f_{i}^{A}f_{j}^{B}}{n}}.$$

The contingency coefficient C as well as the correlation coefficient takes values from the interval 〈0,1〉. If C=0, characters *A*, *B* are independent. Values close to zero indicate weak dependence, and vice versa, values close to 1 indicate strong dependence. The interpretation of the other values of the contingency coefficient is the same as the interpretation of the values of the correlation coefficient. In our case, we calculated a contingency coefficient *C* = 0.302, between personal trust and quality of life, i.e., there is a very moderate degree of association between personal trust and quality of life.

**Figure 4:**
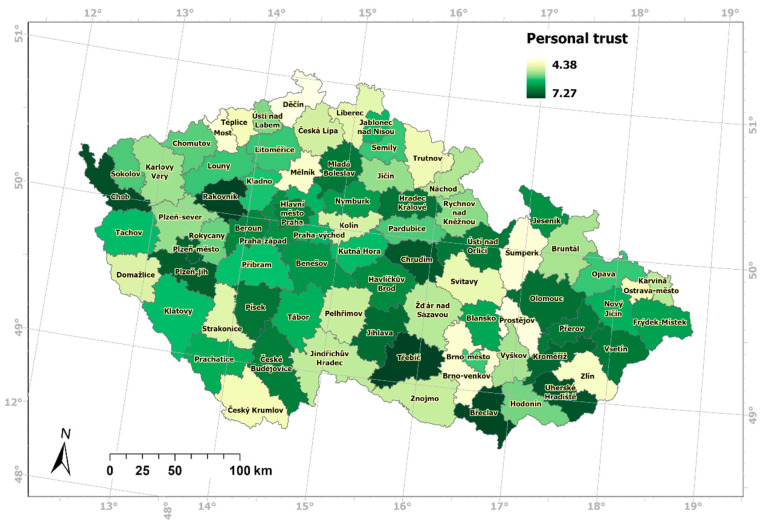
Spatial differentiation of trust in districts of Czechia.

**Figure 5:**
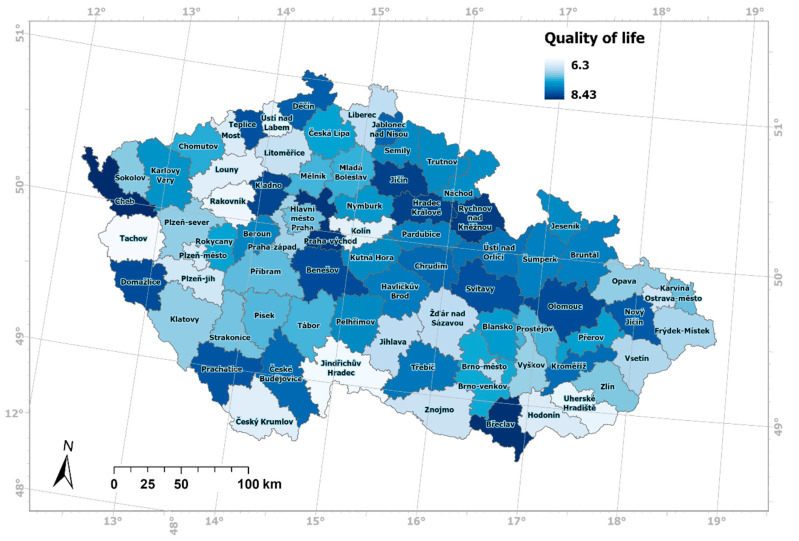
Spatial differentiation of quality of life in districts of Czechia.

The test showed that the values of the quality-of-life index are independent of the values of the personal trust index. Meaning that the quality-of-life index values is not related to the personal trust index values. Using statistical methods, it was confirmed that at the level of Czech districts (Figure 4 and Figure 5), except for the districts with the highest quality-of-life values, social capital as expressed by personal trust is not a predictor of quality of life. The hypothesis of a strong influence of social capital on the quality of life of residents of Czechia was not confirmed. We consider this statement to be important from an epistemological point of view. At the same time, the statement strengthens the specificity of the quality of life in Czechia [15].

Our measurements show that the average value of men’s trust is 5.35, the average value of women’s trust is 5.25 and the overall average value of respondents is 5.30. The geographical approach to social capital research as a predictor of quality of life is based on the recognition that both social capital and quality of life are spatially differentiated. Therefore, we are interested in how the result of our research is differentiated at the district level, i.e., the knowledge that in the Czech Republic social capital expressed by personal trust is not a predictor of quality of life.

On a scale of 0–10, the measured values ranged from 4.38 (Děčín district) to 7.27 (Třebíč district). The quality of life for men was 7.35, and for women 7.48, with the average value of 7.41. On a scale of 0–10, the measured values ranged from 6.30 (Tachov district) to 8.42 (Cheb district). This means that significantly less trust was measured in Czechia than the quality of life. The value of Pearson’s correlation coefficient of trust and quality of life in the districts of Czechia is 0.09, which, according to the verbal expression of correlation values, represents a “very low” correlation, almost zero. The spatial patterns of personal trust values and quality-of-life values are expressed in Figure 4 and Figure 5.

In Table 4, Table 5, Table 6 and Table 7, we list the districts with the highest/lowest personal trust and corresponding quality-of-life values in these districts and the districts with the highest/lowest quality-of-life values and corresponding personal trust values in these districts. A correlation of 0.48, i.e., a medium correlation, can be considered as supporting the claim that personal trust.

This statement holds only for Table 4 with the ten districts with the highest quality-of-life values and the correlation with trust values in them. In the other tables, we see very low correlation (Table 5) and high negative correlation (Table 6 and Table 7).

## 5. Conclusions

In the paper, we focus on social capital, which is considered to be a strong predictor of quality of life or one of the key determinants [17]. We consider the predictor to be an influencing variable for which a change in the value will be reflected in the influenced variable. In the paper, the influencing variable is social capital, and the influenced variable is quality of life. In our understanding of social capital, we draw on Putnam [30], according to whom social capital consists of trust, norms and networks. We consider personal trust to be the most important component of social capital. In the paper, we consider social capital and personal trust to be the same. We formulated a research hypothesis: social capital will have a strong influence on the quality of life of the residents of Czechia, i.e., it will be its predictor. The aim of the research was to outline the epistemology of social capital in terms of quality of life and the description of quality of life, and then to test the validity of the research hypothesis.

We have outlined the epistemology of social capital in terms of quality of life, we have described the concept of quality of life. We measured both personal trust and quality of life in all districts of Czechia so as to satisfy the district quota selection criterion. Residents over 18 years of age were involved in the research by face-to-face interview method and on social networks; both personal trust and quality of life were rated on a scale of 0–10. The personal trust values ranged from 4.38 to 7.27, and the measured quality-of-life values ranged from 6.30 to 8.43. This means that trust is lower than quality of life in the districts of Czechia.

As mentioned above, to verify the validity of the research hypothesis, we used statistical methods to verify the dependence of two qualitative features, namely the chi-square test of independence for the type of contingency table type k × m (79). At the end of the statistical analysis of the research results, we calculated the degree of dependence between the two quantitative features: personal trust and quality of life using the contingency coefficient (79). The contingency coefficient C as well as the correlation coefficient acquires values from the interval 0, 1.

Based on the calculated value of the contingency coefficient between personal trust and quality of life (coefficient C = 0.302), there is a very moderate degree of association between personal trust and quality of life. Additionally, the value of Pearson’s correlation coefficient of trust and quality of life in the districts of Czechia is 0.09, which according to the verbal expression of the correlation values, represents a correlation of “very low”, almost zero. This means that in the districts of Czechia, social capital expressed by personal trust is lower than quality of life. The research hypothesis was not confirmed. This finding holds for all districts of Czechia except for the 10 districts with the highest quality-of-life values, in which the Pearson correlation coefficient between their quality of life and personal trust values is 0.48. In these districts, personal trust is a predictor of quality of life.

The fact that the hypothesis of a strong impact of social capital on the quality of life of the Czech population has not been confirmed—with one exception—is considered to be important from an epistemological point of view. There are two reasons. The first reason is that a general statement, e.g., the labeling of the impact of social capital on quality of life as “robust” [40] or the understanding of social capital as necessary for a high quality of life [1], is not always true. The second reason concerns the aforementioned exception, which consists of ten districts with the highest quality of life. On a scale of 0–10, all these districts have a value of 8.00 and higher. A possible explanation for this fact is that the statement of the position of social capital as a strong predictor of quality of life applies to countries or regions with high quality-of-life values. At the same time, these findings highlight the need for geographical location-oriented research into quality of life. The first key finding of our research in the Czech Republic is the knowledge of the possibility of a higher quality of life at lower values of social capital. The second key finding is the possibility of achieving higher values of social capital at high values of quality of life. This confirms the premise of meaningfulness to improve quality of life as a public policy goal.

## Figures and Tables

**Table 1:** Evolution of personal trust in Czechia from 1999–2017 in % in EVS measures [57].

Trust in Other People (Line in %)		EVS 2017		EVS 2008		EVS 1999	
		Trust in Other People		Trust in Other People		Trust in Other People	
		People Can Be Trusted	One Must Be Careful	People Can Be Trusted	One Must Be Careful	People Can Be Trusted	One Must Be Careful
**Total**		**23**	**77**	**30**	**70**	**24**	**76**
Sex	male	25	75	29	71	23	77
	female	21	79	32	68	25	75
Age	18–29	28	72	33	67	21	79
	30–44	20	80	29	71	22	78
	45–59	20	80	30	70	27	73
	60+	24	76	31	69	24	76
Education	basic	17	83	27	73	20	80
	trained	15	85	32	68	19	81
	SS	26	74	30	70	27	73
	Uni	36	64	33	67	40	60
Population in thous.	Up to 5	24	76	31	69	22	78
	5–19.9	27	73	35	65	20	80
	20–99.9	17	83	26	74	23	77
	100+	24	76	33	67	29	71
Region	Prague	24	76	31	69	30	70
	Czechia	23	77	25	75	23	77
	Morava	22	78	39	61	23	77

**Table 2:** Distribution of districts into groups in the Czech Republic according to the average values of the quality-of-life index and the personal trust index.

	Quality of Life			
Personal Trust	1 Low	2 Medium	3 High	Total
1 (low)	4	12	1	17
2 (medium)	10	32	5	47
3 (high)	5	4	4	13
Total	19	48	10	77

Chi-square: 7.769, *p* = 0.100.

**Table 3:** Distribution of number of observed characters in %.

		Quality of Life		
Personal Trust	Low	Medium	High	Total
Low	5	16	1	22
Medium	13	42	6	61
High	6	5	5	17
Total	25	62	13	100

**Table 4:** Districts with the highest quality of life and corresponding trust values.

Districts with the 10 Highest Quality-of-Life Values (a) and Their Trust Values (b)		
	(a)	(b)
Cheb	8.43	6.73
Břeclav	8.23	7.08
Praha-východ	8.20	6.15
Rychnov n. Kněžnou	8.18	5.82
Hradec Králové	8.10	6.50
Jičín	8.10	5.80
Kladno	8.05	6.05
Benešov	8.00	6.28
Domažlice	8.00	5.45
Olomouc	8.00	6.52
Correlation (a):(b)	0.48	

**Table 5:** Districts with the highest values of trust life and corresponding quality-of-life values.

Districts with the 10 Highest Trust Values (a) and Their Quality-of-Life Values (b)		
	(a)	(b)
Třebíč	7.27	7.67
Rakovník	7.20	6.40
Břeclav	7.08	8.23
Cheb	6.73	8.43
Uh. Hradiště	6.65	6.53
Chrudim	6.64	7.86
Plzeň–jih	6.60	6.80
Plzeň–město	6.55	6.78
Kroměříž	6.54	7.85
Jihlava	6.53	6.94
Correlation (a):(b)	0.11	

**Table 6:** Districts with the lowest quality of life and corresponding trust values.

Districts with the 10 Lowest Quality-of-Life Values (a) and Their Trust Values (b)		
	(a)	(b)
Tachov	6.30	6.10
Jindřichův Hradec	6.36	5.64
Rakovník	6.40	7.20
Uherské Hradiště	6.53	6.65
Ústí nad Labem	6.60	5.87
Kolín	6.67	5.50
Český Krumlov	6.70	5.20
Louny	6.70	6.00
Hodonín	6.77	5.88
Most	6.77	5.00
Correlation (a):(b)	−0.54	

**Table 7:** Districts with the lowest values of trust and corresponding quality-of-life values.

Districts with the 10 Lowest Trust Values (a) and Their Quality-of-Life Values (b)		
	(a)	(b)
Děčín	4.38	7.88
Prostějov	4.46	7.42
Šumperk	4.79	7.64
Brno–venkov	5.00	7.48
Most	5.00	6.77
Mělník	5.08	7.42
Zlín	5.15	7.26
Ostrava	5.16	6.84
Český Krumlov	5.20	6.70
Trutnov	5.27	7.60
Correlation (a):(b)	−0.53	

## Data Availability

Not applicable.
